# Relationship between quantity of IFNT estimated by IFN-stimulated gene expression in peripheral blood mononuclear cells and bovine embryonic mortality after AI or ET

**DOI:** 10.1186/1477-7827-10-21

**Published:** 2012-03-22

**Authors:** Shuichi Matsuyama, Takatoshi Kojima, Satoru Kato, Koji Kimura

**Affiliations:** 1 National Institute of Livestock and Grassland Science, National Agriculture and Food Research Organization, Nasushiobara 329-2793, Japan; 2 National Agricultural Research Center for Western Region, Ohda 694-0013, Japan; 3 Gunma Prefectural Livestock Experiment Station, Maebashi 371-0103, Japan

**Keywords:** Interferon tau, Interferon-stimulated gene 15-kDa protein, Embryonic mortality, Maternal recognition period, Cattle, Peripheral blood mononuclear cells, Corpus luteum, Estrous cycle

## Abstract

**Background:**

Interferon tau (IFNT), which is secreted into the uterine cavity during the maternal recognition period (MRP), is a key factor for establishment of pregnancy. The present study aims to clarify the relationship between the ability of a bovine conceptus to produce IFNT during the MRP and the conceptus's ability to establish pregnancy.

**Methods:**

In the first experiment, IFNT (0, 500, or 1000 micrograms) was administered into the uterine horn ipsilateral to the CL 16 or 17 d after standing estrus, and mRNA levels of IFN-stimulated gene 15-kDa protein (*ISG15*) and *Mx2 *in peripheral blood mononuclear cells (PBMCs) were determined. In the second experiment, we investigated *ISG15 *mRNA expression in PBMCs during the MRP in cattle after either artificial insemination (AI) or embryo transfer (ET).

**Results:**

Intrauterine administration of IFNT stimulated *ISG15 *and *Mx2 *gene expressions in PBMCs in cattle, and there was a positive correlation between the expressions of peripheral markers and the quantity of IFNT administered. In pregnant and normal interestrous interval (< 25 d) cattle (nIEI cattle), expression levels of the *ISG15 *gene showed similar patterns after AI and ET, and *ISG15 *mRNA expression was increased in pregnant cattle but unchanged in nIEI cattle. In contrast, *ISG15 *gene expression in extended interestrous interval (greater than or equal to 25 d) cattle (eIEI cattle) differed after ET compared with AI. In eIEI cattle after ET, *ISG15 *gene expression increased, such that the value on day 18 was intermediate between those of pregnant and nIEI cattle. In eIEI cattle after AI, *ISG15 *gene expression did not increase throughout the observation period.

**Conclusions:**

The results of the current study indicate that the quantity of conceptus-derived IFNT can be estimated by measuring *ISG15 *mRNA levels in PBMCs from cattle. Using this approach, we demonstrate that *ISG15 *gene expression during the MRP in eIEI cattle differed after ET compared with AI. In addition, the modest increase in *ISG15 *gene expression in eIEI cattle after ET suggests that late embryo losses were due to delayed or insufficient growth of the conceptus during the MRP in cattle.

## Background

In most mammalian species, an embryo must signal its presence to the mother to establish a successful pregnancy. The signaling molecule in ruminants is interferon tau (IFNT), which is secreted from the trophectoderm of the conceptus beginning at the blastocyst stage [1] and increases with elongation of the conceptus [2-5]. The production of IFNT peaks just before the conceptus attaches to the uterine epithelium (implantation) [6,7]; after implantation, the secretion of IFNT attenuates. IFNT interacts with its receptor on the uterine endometrium and modulates the secretion of prostaglandin F_2α _from the endometrium, thereby maintaining the function of the corpus luteum (CL) [8-12]. Consequently, the IFNT level during the maternal recognition period (MRP) is considered to be important for successful establishment of pregnancy in ruminants [13-15].

For many years, IFNT had been thought to be secreted exclusively within the uterus and to not enter the peripheral circulation. However, some reports have suggested that the expression of various interferon-stimulated genes is upregulated in peripheral blood cells in pregnant cattle during the MRP [16-18]. Recent studies clearly demonstrate that a portion of the IFNT secreted into the uterine cavity enters the uterine vein and directly upregulates the expression of IFN-stimulated genes in both peripheral blood cells and the CL [19,20]. This upregulation of IFN-stimulated gene expression in peripheral blood is a potential target for new methods of early diagnosis of pregnancy. For example, mRNA levels of *Mx*, an IFN-stimulated gene, are increased in the peripheral blood mononuclear cells (PBMCs) of pregnant ewes from 15 through 30 d after insemination [17]. In addition, gene expression levels of *Mx1, Mx2*, and interferon-stimulated gene 15-kDa protein (*ISG15*) during the MRP are greater in pregnant compared with bred, nonpregnant cows [18]. A reliable method of measuring IFN-stimulated gene expression for early pregnancy detection potentially could be applied as early as 18 d after insemination of heifers [21].

In addition to its use for early pregnancy detection, a method measuring IFN-stimulated gene expression might be effective for evaluating embryo survival [16]. Embryonic mortality is a key factor causing pregnancy loss in cattle [22-26]. In classifying pregnancy loss according to luteolysis and interestrous intervals, a return to estrus before day 24 indicates 'early embryonic loss,' that occurring between days 24 to 50 is referred to as 'late embryonic loss,' and pregnancy loss detected after day 50 is characterized as 'fetal loss' [27]. Losses of pregnancy are characterized by early embryonic death, which occurs between days 8 and 16 of gestation [22,23]. Moreover, embryonic death in the case of late embryonic loss has been suggested to occur beyond day 16 of gestation, because the lifespan of the CL is extended in this case [27]. Indeed, the expression levels of *ISG15 *mRNA during the MRP differ between cattle that have undergone early compared with late embryonic loss after artificial insemination (AI) [16]. Most of the studies on embryo mortality involve cattle that have undergone AI, and the incidence and manner of pregnancy loss after embryo transfer (ET) might differ from those after AI, for which failure of fertilization is included in the calculation of pregnancy loss.

Therefore, we first sought to confirm the positive relationship between IFNT levels in the uterus and ISGs mRNA expression in the PBMCs of cattle. To demonstrate the relationship between production of IFNT by a bovine conceptus and embryonic mortality, we investigated *ISG15 *mRNA expression in PBMCs during the MRP of cattle after either AI or ET.

## Methods

### Animals

All cattle were fed a grass silage-based diet *ad libitum*. All experimental procedures involving animals were approved by the committee for the Care and Use of Experimental Animals at the National Institute of Livestock and Grassland Science.

### Experiment 1: Relationship between the quantity of IFNT administered into the uterus and the expressions of *ISG15 *and *Mx2 *mRNAs in PBMCs

Six female multiparous crossbred (Japanese Black × Holstein-Friesian) beef cows weighing 610 to 730 kg were used in a randomized crossover design. Estrus was synchronized among these cows by providing each with two injections of prostaglandin F_2α _(0.5 mg i.m.; cloprostenol, Nihon Zenyaku Kogyo, Fukushima, Japan) separated by an interval of 14 d.

Recombinant bovine IFNT (bTP-509A) produced by *E. coli*. [28] was diluted with PBS to a concentration of 0, 1, or 2 mg/ml; BSA (Nacalai Tesque, Kyoto, Japan) was added as needed to bring the total protein concentration of the IFNT-containing mixture to 2 mg/ml. Aliquots (500 μl each) of the IFNT solutions were enclosed in plastic semen straws for administration into the uterine horn ipsilateral to the CL 16 or 17 d after standing estrus.

For determination of *ISG15 *and *Mx2 *mRNA expressions, blood samples (5 ml) were obtained at 2 h before and 0, 2, 4, 6, 8, 10, 12, 16, 20, and 24 h after administration of IFNT. Blood samples were collected through a 16-gauge jugular catheter (Unitika, Osaka, Japan) that had been implanted on the day before IFNT administration.

### Experiment 2: *ISG15 *gene expression during the MRP after AI or ET

Dried primiparous or multiparous cattle weighing 291 to 755 kg each either underwent AI at standing estrus (d 0) or received a frozen-thawed in-vivo-derived embryo into the uterine horn ipsilateral to the CL at 7 d after standing estrus. In order to compare the production of IFNT by a bovine conceptus among the pregnancy status, cattle after AI or ET were classified into 3 groups: pregnant cattle; extended interestrous interval (≥ 25 d) cattle (eIEI cattle); and normal interestrous interval (< 25 d) cattle (nIEI cattle) [29]. In this experiment, all eIEIs cattle (AI; n = 9, ET; n = 11) were used because the incidence of late embryonic loss was very low [27,30,31]. On the other hand, pregnant (AI; n = 13, ET; n = 16) and nIEIs (AI; n = 19, ET; n = 17) cattle were selected randomly. Untreated cycling cattle (n = 15) were used as controls.

Jugular blood samples were collected as described at 7, 16, 18, 21, and 25 d after standing estrus for analysis of ISG15 mRNA expression and at 7, 12, 16, 18, 21, and 25 d after standing estrus for analysis of plasma progesterone (P_4_) concentrations. Pregnancy was diagnosed by transrectal ultrasonography at 30 to 50 d after standing estrus.

### Embryos

Embryos were collected nonsurgically from superovulated Japanese Black cattle on day 7 after standing estrus, as described previously [32]. Embryos staged at grade 1 or 2 blastocysts according to the IETS manual [33] were cryopreserved by using ethylene glycol as a cryoprotectant [34]. After being thawed, the embryos were cultured in TCM199 medium (Invitrogen, Carlsbad, CA, USA) supplemented with 10% FBS (ICN Biomedicals, Aurora, OH, USA) at 38.5°C in 5% CO_2_, 5% O_2_, 90% N_2_. After 6 h of culture, the quality of viable embryos was reevaluated and only grade 1 or 2 embryos were used for ET.

### Blood sample processing

Blood samples for the determination of plasma P_4 _concentrations were collected into heparin-containing tubes (Terumo, Tokyo, Japan), and plasma was separated by centrifugation at 4°C for 30 min and stored at -30°C until assayed. Blood samples for RNA extraction were collected into tubes containing potassium EDTA (Terumo). Samples were centrifuged at 4°C for 30 min, and buffy coat fractions were collected. Contaminating red blood cells were removed by hemolysis, and PBMCs were isolated. The PBMCs were resuspended in TRIzol reagent (Invitrogen) and stored at -80°C until analysis.

### RNA extraction, cDNA synthesis, and quantitative real-time RT-PCR

Total RNA was extracted from PBMC samples by using TRIzol (Invitrogen) in accordance with the manufacturer's protocol. Single-stranded cDNA was synthesized from 1 μg of RNA by using SuperScript II Reverse Transcriptase (Invitrogen) with oligo-dT_12-18 _primer (Invitrogen) in accordance with the manufacturer's instructions. The resulting cDNA was used as a template for quantitative real-time reverse transcription-polymerase chain reaction (RT-PCR) analysis. The sequences of primers and probes for *ISG15, Mx2 *and *GAPDH *are listed in Table 1. Each reaction mixture consisted of cDNA, forward and reverse primers (200 to 500 nM each), probes (100 to 300 nM each), Brilliant II QPCR Master Mix (Agilent Technologies, Palo Alto, CA, USA), and nuclease-free water in a total reaction volume of 20 μl. Thermocycling conditions included initial sample incubation at 50°C for 2 min, then 95°C for 10 min, followed by 50 cycles of 30 sec at 95°C followed by 90 sec at 60°C. Serial dilutions of plasmid containing *ISG15, Mx2 *or *GAPDH *were used as standards. Results are reported as the *n*-fold difference relative to a calibrator cDNA (i.e., experiment 1: transcript of sample at 2 h before IFNT administration; experiment 2: transcript of sample on day 7 after standing estrus) after normalization of the transcript signals to the endogenous control *GAPDH*.

**Table 1:** Oligonucleotide primers and probes used for real-time RT-PCR analysis

Gene	**GenBank accession no**.		Sequence	Position (nucleotide no.)
ISG15	NM_174366	Forward primer	5' GGGACCTGACGGTGAAGATG 3'	67-86
		Reverse primer	5' GAAAGCAGGCACATTGATCTTCT 3'	160-182
		*Taq*Man probe	5' TCCTGGTGCCTCTGAGGGACTCCAT 3'	103-127
Mx2	NM_173941	Forward primer	5' AAATCACCTACCGCAACATTACG 3'	772-794
		Reverse primer	5' GCCAAGTCCATTCCCAGCTA 3'	853-872
		*Taq*Man probe	5' ATGTTCTGGGCTCTCCGA 3'	833-850
GAPDH	U85042	Forward primer	5' GGCACAGTCAAGGCAGAGAAC 3'	238-258
		Reverse primer	5' GGATCTCGCTCCTGGAAGATG 3'	291-311
		*Taq*Man probe	5' CATCAATGGAAAGGCCA 3'	270-286

### P_4 _assay

Plasma P_4 _concentrations were determined by using a double-antibody enzyme immunoassay as described previously [35]. Briefly, 100 μl of plasma sample was extracted twice with 2 ml diethyl ether in a glass tube. After evaporation of the diethyl ether, the residue was dissolved in 500 μl of assay buffer (0.01 M PBS, pH 7.0, containing 1% [w/v] BSA) and used for the assay. Microtiter plates were coated with anti rabbit γ globulin goat serum (50 ng/well in 0.1 M carbonate buffer, pH 9.6; Cappel, MP Biomedicals, Solon, Ohio, USA) as the priming antiserum. A rabbit antiserum raised against 11α-hydroxy-progesterone-HS-BSA (a gift from Dr. Takenouchi) was diluted to 1:800,000. Progesterone-3-CMO-HRP (Cosmo Bio, Tokyo, Japan) diluted to 1:500,000 was used as the steroid-enzyme conjugate for visualization of the signal. Assay sensitivity was 0.1 ng/ml for 100-μl plasma samples; therefore, a sample yielding a signal below this threshold was assigned a value of 0.1 ng/ml. The intra- and interassay coefficients of variation were 7.8% at 4.8 ng/ml and 2.8% at 5.0 ng/ml, respectively.

### Statistical analysis

Statistically significant differences (*P *< 0.05) in relative *ISG15 *and *Mx2 *mRNA expression levels were determined by using one-way ANOVA for repeated measures followed by Dunnett's test. Regression analysis was used to evaluate the relationship between the levels of relative *ISG15 *and *Mx2 *mRNA expressions at 4 h after administration of IFNT and the dose (μg/kg body weight) of IFNT administered into the uterus.

The estrus was decided by standing behavior or sharp decline in plasma P_4 _values. In addition, untreated cycling cattle were used as controls. Statistically significant differences (*P *< 0.05) in relative *ISG15 *mRNA expression and plasma P_4 _concentrations on days 16, 18, and 21 were determined by using one-way ANOVA followed by the Tukey HSD test within each day, and those on day 25 were determined by using Student's *t *test. Mean estrous cycle length in eIEI cattle were analyzed by using Student's *t *test.

## Results

### Relationship between the quantity of IFNT administered into the uterus and the *ISG15 *and *Mx2 *mRNA expression levels in PBMCs

**Figure 1:**
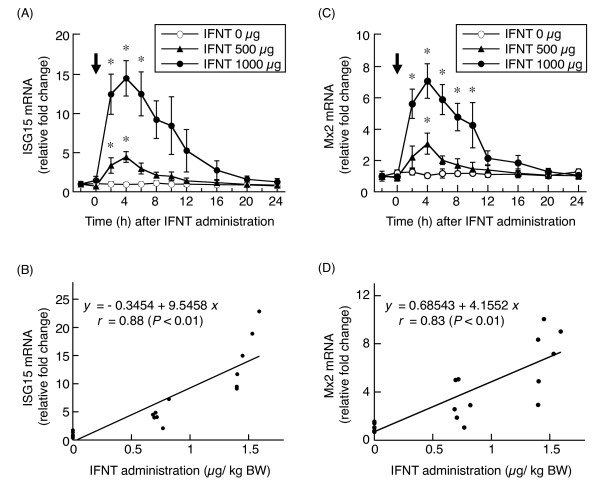
***ISG15 *and *Mx2 *mRNA expressions after intrauterine administration of IFNT**. *ISG15 *(A) and *Mx2 *(C) mRNA expressions (mean ± S.E.M.) in PBMCs of cattle after intrauterine administration of IFNT (arrow; 0, 500, or 1000 μg); data are presented as relative fold change from the amount at 2 h before IFNT administration. *, *P *< 0.05 compared with value at 2 h before IFNT administration. Regression line of *ISG15 *(B) and *Mx2 *(D) mRNA expressions at 4 h after IFNT administration and concentration of IFNT administered into the uterus.

Intrauterine administration of IFNT significantly (*P *< 0.05) increased *ISG15 *and *Mx2 *mRNA expressions in the PBMCs of cattle relative to pretreatment values. *ISG15 *(Figure 1A) and *Mx2 *(Figure 1C) mRNA levels began to increase at 2 h after administration of IFNT, peaked at 4 h, and subsequently decreased and returned to the basal. The *ISG15 *(*r *= 0.88, *P *< 0.01) and *Mx2 *(*r *= 0.83, *P *< 0.01) mRNA expression levels at 4 h after IFNT treatment were positively correlated with the dose of IFNT administered into the uterus (Figure 1B and 1D).

### *ISG15 *mRNA expression during the MRP after AI or ET

**Figure 2:**
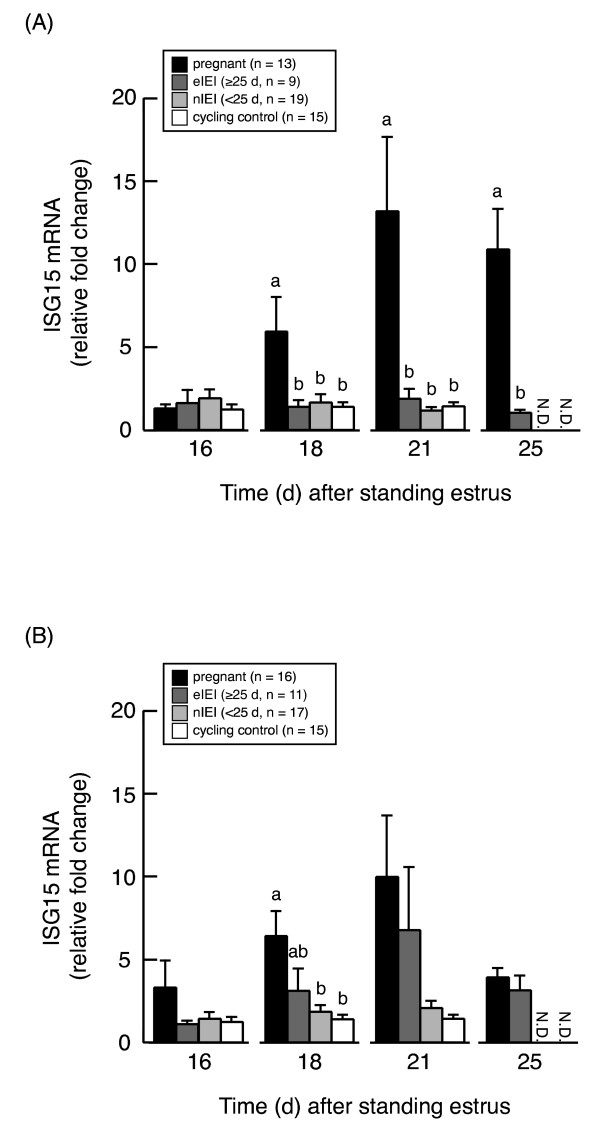
***ISG15 *mRNA expressions in each pregnancy status of cattle after AI or ET**. *ISG15 *mRNA expression (mean ± S.E.M.) on days 16, 18, 21, and 25 after standing estrus in pregnant, extended interestrous interval (≥ 25 d, eIEI), and normal interestrous interval (< 25 d, nIEI) cattle after AI (A) or ET (B). Data are presented as relative fold change from value on day 7; comparisons were made within each day. Different letters indicate a significant (*P *< 0.05) difference between values.

Among cattle that underwent AI, the average *ISG15 *mRNA expression in PBMCs on days 18, 21, and 25 was significantly (*P *< 0.05) higher in pregnant cattle (n = 13) than in any other group on the same day (Figure 2A). *ISG15 *mRNA expression on days 18 and 21 after AI did not differ among eIEI (n = 9), nIEI (n = 19), and cycling control cattle (n = 15).

Among cattle that underwent ET, the mean *ISG15 *mRNA on day 18 was significantly (*P *< 0.05) higher in pregnant cattle (n = 16) than in nIEI (n = 17) and cycling control cattle (n = 15) (Figure 2B). *ISG15 *mRNA expression on day 18 after ET in eIEI cattle (n = 11) was intermediate between the value for pregnant cattle and both of those for nIEI and cycling control cattle, although none of these differences reached significance.

### Changes in plasma P_4 _concentrations after AI or ET

**Figure 3:**
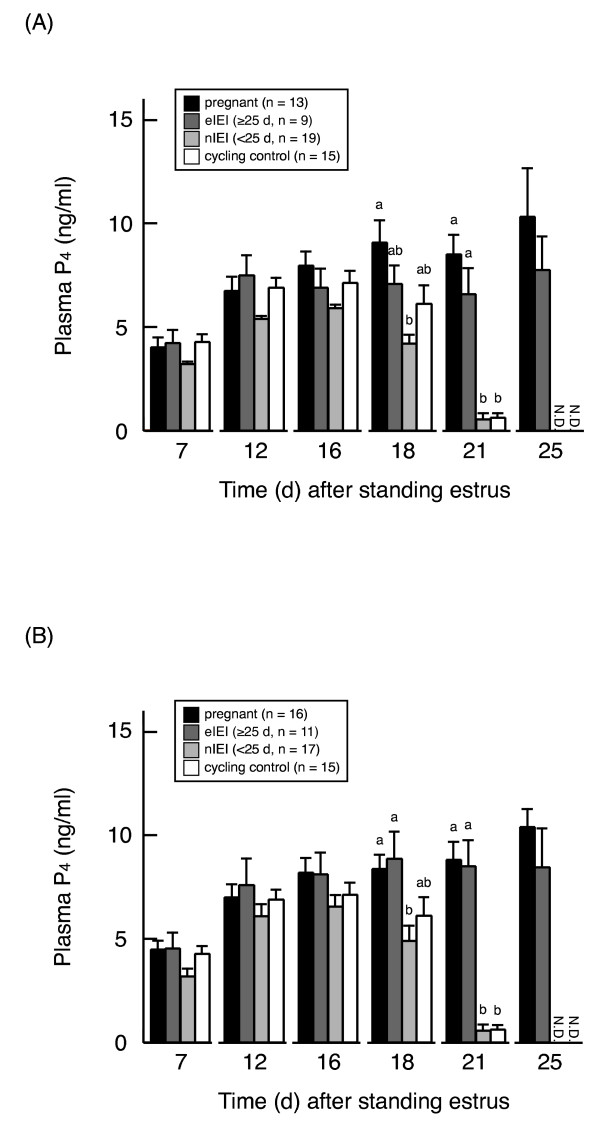
**Plasma progesterone concentrations in each pregnancy status of cattle after AI or ET**. Plasma progesterone concentration (mean ± S.E.M.) on days 7, 12, 16, 18, 21, and 25 after standing estrus in pregnant, extended interestrous interval (≥ 25 d, eIEI), and normal interestrous interval (< 25 d, nIEI) cattle after AI (A) or ET (B). Comparisons were made within each day. Different letters indicate a significant (*P *< 0.05) difference between values.

After AI or ET, plasma P_4 _concentration on days 7, 12, 16, and 25 did not differ between pregnant and eIEI cattle (Figure 3). On day 18 after AI, nIEI cattle had significantly lower plasma P_4 _concentrations than pregnant cattle, and numerically but not significantly lower concentrations than eIEI and control cattle. On day 18 after ET, plasma P_4 _concentrations in nIEI cattle were significantly lower than those in both pregnant and eIEI cattle. Furthermore, plasma P_4 _concentration on day 21 after AI or ET was significantly (*P *< 0.05) lower in nIEI and control cattle than in pregnant and eIEI cattle.

### Return to estrus in eIEI cattle after AI or ET

In eIEI cattle after AI and ET, the mean length of the estrous cycle was 35.1 ± 3.8 and 39.5 ± 1.8 d, respectively (Table 2); these values did not differ significantly. The distribution of return to estrus in eIEI cattle was 25 to 29 d (44.4% of the group) for AI and 40 to 44 d (36.4%) for ET.

**Table 2:** Estrous cycle length in eIEI cattle after AI or ET

		Estrous cycle length (d)^a^					
	Group mean ± SEM	25-29	30-34	35-39	40-44	45-49	50-
AI (n = 9)	35.1 ± 3.8	44.4% (4)	11.1% (1)	22.2% (2)	0% (0)	11.1% (1)	11.1% (1)
ET (n = 11)	39.5 ± 1.8	9.1% (1)	18.2% (2)	27.3% (3)	36.4% (4)	9.1% (1)	0% (0)

^a^Values are given as % of group (n).

## Discussion

Although IFNT is a key factor for the first step of establishment of pregnancy, whether the ability of a bovine embryo to produce IFNT reflects its capacity to establish pregnancy has not been clarified. Measuring the expression of IFN-stimulated genes has been suggested as a possible method for assessing embryo survival as well as for early detection of pregnancy [16]. However, the correlation between the quantity of IFNT secretion excreted into the uterus and the levels of IFN-stimulated gene expression in PBMCs had not been determined previously. Therefore, we measured the levels of *ISG15 *and *Mx2 *mRNAs in bovine PBMCs after intrauterine administration of recombinant IFNT. Intrauterine administration of IFNT stimulated *ISG15 *and *Mx2 *gene expressions in PBMCs in cattle, and there were positive correlations between the expressions of peripheral markers and the quantity of IFNT administered. In addition, the correlation coefficient between *Mx2 *and IFNT (r = 0.83) was slightly lower than that between *ISG15 *and IFNT (r = 0.88). These results suggest that the quantity of conceptus-produced IFNT could be monitored by measuring *ISG15 *and *Mx2 *mRNA expressions in PBMCs, thereby providing a powerful noninvasive tool to evaluate embryonic mortality.

The present study is the first to report changes in *ISG15 *mRNA expression in PBMCs in cattle after ET. In pregnant and nIEI cattle, *ISG15 *gene expression after ET followed a similar pattern as the expression after AI. In the current study, *ISG15 *mRNA expression in pregnant cattle was increased during the MRP as reported previously [18,21]. In cattle, a portion of the IFNT secreted by the developing conceptus during the MRP enters into the uterine vein and directly upregulates the expression of IFN-stimulated genes in peripheral blood cells [20]. The present study revealed that the amount of IFNT administered into the uterus is positively correlated with *ISG15 *mRNA expression in PBMCs. In addition, some studies have demonstrated that the quantity of IFNT production parallels the degree of trophoblastic elongation in cattle [2-4] and sheep [5]. Therefore, the magnitude of increase in *ISG15 *mRNA expression in PBMCs likely reflects the amounts of conceptus elongation and IFNT secretion.

nIEI cattle lacked any increase in *ISG15 *mRNA expression during the MRP. Previous investigations [22,36] indicate that fertilization rates are high (on the order of 90%) in cattle, suggesting that embryonic death is responsible for the majority of reproductive failure. Moreover, the greatest incidence of embryonic death is considered to occur between days 8 and 16 after insemination [22]. These previous findings might well explain the results of the current study, such that early embryonic loss (that is, before the MRP) would account for the observed lack of increase in *ISG15 *gene expression in nIEI cattle.

Unlike those in pregnant and nIEI cattle, the changes in *ISG15 *gene expression in eIEI cattle differed depending on whether the animal underwent AI or ET. In eIEI cattle after ET, *ISG15 *gene expression increased somewhat, with the value on day 18 intermediate to those of pregnant and nIEI cattle. This result suggests that even if the conceptus can secrete IFNT during the MRP, pregnancy is not always established. As mentioned earlier, the *ISG15 *mRNA expression in PBMCs reflects not only the quantity of conceptus-produced IFNT but also the degree of conceptus growth. Therefore, it is possible that the comparatively limited increase in *ISG15 *gene expression in eIEI cattle results from delayed conceptus growth, as previously suggested to occur in dairy cows [16]. As another possibility, the comparatively low IFNT production in eIEI cattle suggests insufficient growth and low viability of conceptuses, which consequently die beyond the MRP. In addition, because IFNT acts on the endometrium to regulate genes important for uterine receptivity and conceptus growth [37-39], the comparatively low IFNT quantity in eIEI cattle might be insufficient to induce endometrial functions necessary for conceptus growth.

Moreover, in eIEI cattle after ET, there was no correlation between *ISG15 *mRNA expression and plasma P_4 _on the same day (data not shown). In addition, plasma P_4 _concentrations during the MRP after ET were not different between eIEI and pregnant cattle, indicating that there was no difference on the CL function. Taken together, these findings suggest that CL regression is inhibited regardless of the quantity of IFNT. Further study is needed to clarify the mechanism of beginning of CL regression in eIEI cattle after ET.

Since ISG15 responds to not only various types of IFNs but also viral infection [40,41], increase of *ISG15 *gene expression found in this study might attribute to viral infection. Moreover, it was reported that uterine infections in cows can extend IEIs independent of pregnancy status [42,43]. Therefore, it could not deny the possibility that the elevation of *ISG15 *gene expression was induced by the subclinical uterine infection in eIEI cattle after ET, although the clinical uterine infection was not detected in the present study. The effect of the subclinical uterine infection on the IEIs and *ISG15 *gene expression must be investigated in future.

In contrast to the scenario after ET, *ISG15 *gene expression did not increase during the MRP of eIEI cattle after AI, and the values did not differ compared with those in nIEI and cycling control cattle. The reason for the invariable level of *ISG15 *gene expression is unclear, but perhaps our method for measuring *ISG15 *mRNA expression in PBMCs was insufficiently sensitive to detect slight increases. In addition, plasma P_4 _concentrations did not differ between eIEI and pregnant cattle after AI. These results suggest that CL function was maintained during the MRP in eIEI cattle after AI and that estrous cycle length was extended even though embryonic loss occurred before the MRP. Although it was not clarified how estrous cycle length was extended in eIEI cattle after AI, we speculate that embryonic death in eIEI cattle after AI occurred somewhat later than in nIEI cattle after AI. In the present study, the most frequent range of return to estrus in eIEI cattle after AI was 25 to 29 d (44.4%). This slightly extended interestrous period in many eIEI cattle after AI supports our speculation that later embryonic death in eIEI than nIEI cattle is associated with inhibition of CL regression and delay of return to estrus. Moreover, regardless of breeding method, the number of days until return to estrus in eIEI cattle was not correlated with *ISG15 *mRNA level during the MRP (data not shown). Accordingly, the distribution of estrous cycle length might be influenced by the stage of follicular development at the beginning of CL regression rather than by the quantity of IFNT secreted by the conceptus.

## Conclusion

The results of the present study indicate that the quantity of conceptus-derived IFNT in cattle can be estimated by measuring *ISG15 *gene expression in PBMCs. Using this approach, we demonstrate that *ISG15 *gene expression during the MRP in eIEI cattle differed depending on whether cows underwent AI or ET. In addition, the slight increase in *ISG15 *gene expression in eIEI cattle after ET suggests that late embryonic losses were due to delayed or insufficient conceptus growth during the MRP.

## Abbreviations

AI: Artificial insemination; CL: Corpus luteum; ET: Embryo transfer; IFNT: Interferon tau; ISG15: Interferon-stimulated gene 15-kDa protein; MRP: Maternal recognition period; PBMC: Peripheral blood mononuclear cell; PBS: Phosphate buffered saline; BSA: Bovine serum albumin; P_4: _Progesterone; FBS: Fetal bovine serum; RT-PCR: Real-time reverse transcription-polymerase chain reaction; GAPDH: Glyceraldehyde-3-phosphate dehydrogenase; eIEI cattle: Cattle with extended interestrous interval (≥ 25 d); nIEI cattle: Cattle with normal interestrous interval (< 25 d).

## Competing interests

The authors declare that they have no competing interests.

## Authors' contributions

SM carried out quantitative real-time RT-PCR assays, P_4 _assays, AI, ET, and statistical analysis; participated in the design of the study; and drafted the manuscript. TK performed quantitative real-time RT-PCR assays and participated in the design of the study. SK carried out AI and ET and participated in the design of the study. KK performed administration of IFNT into the uterus, recovery of embryos, AI, and ET; participated in the design of the study; and helped to draft the manuscript. All authors read and approved the final manuscript.

## References

[B1] Hernandez-Ledezma JJ Sikes JD Murphy CN Watson AJ Schultz GA Roberts RM Expression of bovine trophoblast interferon in conceptuses derived by in vitro techniques Biol Reprod 1992 47 3 374 380 10.1095/biolreprod47.3.374 1511091

[B2] Garrett JE Geisert RD Zavy MT Morgan GL Evidence for maternal regulation of early conceptus growth and development in beef cattle J Reprod Fertil 1988 84 2 437 446 10.1530/jrf.0.0840437 3199361

[B3] Mann GE Fray MD Lamming GE Effects of time of progesterone supplementation on embryo development and interferon-tau production in the cow Vet J 2006 171 3 500 503 10.1016/j.tvjl.2004.12.005 16624716

[B4] Mann GE Lamming GE Relationship between maternal endocrine environment, early embryo development and inhibition of the luteolytic mechanism in cows Reproduction 2001 121 1 175 180 10.1530/rep.0.1210175 11226041

[B5] Nephew KP Cardenas H McClure KE Ott TL Bazer FW Pope WF Effects of administration of human chorionic gonadotropin or progesterone before maternal recognition of pregnancy on blastocyst development and pregnancy in sheep J Anim Sci 1994 72 2 453 458 8157530 10.2527/1994.722453x

[B6] Bartol FF Roberts RM Bazer FW Lewis GS Godkin JD Thatcher WW Characterization of proteins produced in vitro by periattachment bovine conceptuses Biol Reprod 1985 32 3 681 693 10.1095/biolreprod32.3.681 3995135

[B7] Roberts RM Cross JC Leaman DW Unique features of the trophoblast interferons Pharmacol Ther 1991 51 3 329 345 10.1016/0163-7258(91)90064-S 1724322

[B8] Hansen TR Kazemi M Keisler DH Malathy PV Imakawa K Roberts RM Complex binding of the embryonic interferon, ovine trophoblast protein-1, to endometrial receptors J Interferon Res 1989 9 2 215 225 10.1089/jir.1989.9.215 2523944

[B9] Spencer TE Bazer FW Ovine interferon tau suppresses transcription of the estrogen receptor and oxytocin receptor genes in the ovine endometrium Endocrinology 1996 137 3 1144 1147 10.1210/en.137.3.1144 8603586

[B10] Spencer TE Becker WC George P Mirando MA Ogle TF Bazer FW Ovine interferon-tau inhibits estrogen receptor up-regulation and estrogen-induced luteolysis in cyclic ewes Endocrinology 1995 136 11 4932 4944 10.1210/en.136.11.4932 7588227

[B11] Thatcher WW Meyer MD Danet-Desnoyers G Maternal recognition of pregnancy J Reprod Fertil Suppl 1995 49 15 28 7623310

[B12] Vallet JL Bazer FW Fliss MF Thatcher WW Effect of ovine conceptus secretory proteins and purified ovine trophoblast protein-1 on interoestrous interval and plasma concentrations of prostaglandins F-2α and E and of 13,14-dihydro-15-keto prostaglandin F-2α in cyclic ewes J Reprod Fertil 1988 84 2 493 504 10.1530/jrf.0.0840493 3199368

[B13] Hansen TR Austin KJ Perry DJ Pru JK Teixeira MG Johnson GA Mechanism of action of interferon-tau in the uterus during early pregnancy J Reprod Fertil Suppl 1999 54 329 339 10692865

[B14] Roberts RM Cross JC Leaman DW Interferons as hormones of pregnancy Endocr Rev 1992 13 3 432 452 1385108 10.1210/edrv-13-3-432

[B15] Spencer TE Burghardt RC Johnson GA Bazer FW Conceptus signals for establishment and maintenance of pregnancy Anim Reprod Sci 2004 82-83 537 550 15271478 10.1016/j.anireprosci.2004.04.014

[B16] Han H Austin KJ Rempel LA Hansen TR Low blood ISG15 mRNA and progesterone levels are predictive of non-pregnant dairy cows J Endocrinol 2006 191 2 505 512 10.1677/joe.1.07015 17088421

[B17] Yankey SJ Hicks BA Carnahan KG Assiri AM Sinor SJ Kodali K Stellflug JN Ott TL Expression of the antiviral protein Mx in peripheral blood mononuclear cells of pregnant and bred, non-pregnant ewes J Endocrinol 2001 170 2 R7 R11 10.1677/joe.0.170R007 11479146

[B18] Gifford CA Racicot K Clark DS Austin KJ Hansen TR Lucy MC Davies CJ Ott TL Regulation of interferon-stimulated genes in peripheral blood leukocytes in pregnant and bred, nonpregnant dairy cows J Dairy Sci 2007 90 1 274 280 10.3168/jds.S0022-0302(07)72628-0 17183095

[B19] Bott RC Ashley RL Henkes LE Antoniazzi AQ Bruemmer JE Niswender GD Bazer FW Spencer TE Smirnova NP Anthony RV Hansen TR Uterine vein infusion of interferon tau (IFNT) extends luteal life span in ewes Biol Reprod 2010 82 4 725 735 10.1095/biolreprod.109.079467 20042537

[B20] Oliveira JF Henkes LE Ashley RL Purcell SH Smirnova NP Veeramachaneni DN Anthony RV Hansen TR Expression of interferon (IFN)-stimulated genes in extrauterine tissues during early pregnancy in sheep is the consequence of endocrine IFN-tau release from the uterine vein Endocrinology 2008 149 3 1252 1259 18063687 10.1210/en.2007-0863

[B21] Green JC Okamura CS Poock SE Lucy MC Measurement of interferon-tau (IFN-tau) stimulated gene expression in blood leukocytes for pregnancy diagnosis within 18-20d after insemination in dairy cattle Anim Reprod Sci 2010 121 1-2 24 33 20554404 10.1016/j.anireprosci.2010.05.010

[B22] Diskin MG Sreenan JM Fertilization and embryonic mortality rates in beef heifers after artificial insemination J Reprod Fertil 1980 59 2 463 468 10.1530/jrf.0.0590463 7431304

[B23] Dunne LD Diskin MG Sreenan JM Embryo and foetal loss in beef heifers between day 14 of gestation and full term Anim Reprod Sci 2000 58 1-2 39 44 10.1016/S0378-4320(99)00088-3 10700643

[B24] Roche JF Bolandl MP McGeady TA Reproductive wastage following artificial insemination of heifers Vet Rec 1981 109 18 401 404 10.1136/vr.109.18.401 7340073

[B25] Silke V Diskin MG Kenny DA Boland MP Dillon P Mee JF Sreenan JM Extent, pattern and factors associated with late embryonic loss in dairy cows Anim Reprod Sci 2002 71 1-2 1 12 10.1016/S0378-4320(02)00016-7 11988367

[B26] Horan B Mee JF Rath M O'Connor P Dillon P The effect of strain of Holstein-Friesian cow and feeding system on reproductive performance in seasonal calving milk production systems Animal Sci 2004 79 453 467

[B27] Humblot P Use of pregnancy specific proteins and progesterone assays to monitor pregnancy and determine the timing, frequencies and sources of embryonic mortality in ruminants Theriogenology 2001 56 9 1417 1433 10.1016/S0093-691X(01)00644-6 11768808

[B28] Imakawa K Anthony RV Kazemi M Marotti KR Polites HG Roberts RM Interferon-like sequence of ovine trophoblast protein secreted by embryonic trophectoderm Nature 1987 330 6146 377 379 10.1038/330377a0 2446135

[B29] Plante C Hansen PJ Martinod S Siegenthaler B Thatcher WW Pollard JW Leslie MV Effect of intrauterine and intramuscular administration of recombinant bovine interferon α_1 _on luteal lifespan in cattle J Dairy Sci 1989 72 7 1859 1865 10.3168/jds.S0022-0302(89)79304-8 2778169

[B30] Santos JE Thatcher WW Chebel RC Cerri RL Galvao KN The effect of embryonic death rates in cattle on the efficacy of estrus synchronization programs Anim Reprod Sci 2004 82-83 513 535 15271477 10.1016/j.anireprosci.2004.04.015

[B31] Diskin MG Murphy JJ Sreenan JM Embryo survival in dairy cows managed under pastoral conditions Anim Reprod Sci 2006 96 3-4 297 311 10.1016/j.anireprosci.2006.08.008 16963203

[B32] Kimura K Hirako M Iwata H Aoki M Kawaguchi M Seki M Successful superovulation of cattle by a single administration of FSH in aluminum hydroxide gel Theriogenology 2007 68 4 633 639 10.1016/j.theriogenology.2007.02.016 17583782

[B33] Stringfellow DA Seidel SM Manual of the International Embryo Transfer Society: A procedural guide and general information for the use of embryo transfer technology emphasizing sanitary procedures 1998 3 Savoy, IL: International Embryo Transfer Society

[B34] Dochi O Yamamoto Y Saga H Yoshiba N Kano N Maeda J Miyata K Yamauchi A Tominaga K Oda Y Nakashima T Inohae S Direct transfer of bovine embryos frozen-thawed in the presence of propylene glycol or ethylene glycol under on-farm conditions in an integrated embryo transfer program Theriogenology 1998 49 5 1051 1058 10.1016/S0093-691X(98)00053-3 10732112

[B35] Takenouchi N Oshima K Shimada K Takahashi M The development of a sensitive enzyme immunoassay for the determination of estrone and estradiol-17β in bovine blood plasma based on the same homologous combination with antiserum and steroid-enzyme conjugate J Vet Med Sci 2004 66 11 1315 1321 10.1292/jvms.66.1315 15585942

[B36] Henricks DM Hill JR Dickey JF Plasma ovarian hormone levels and fertility in beef heifers treated with melengestrol acetate (MGA) J Anim Sci 1973 37 5 1169 1175 4758022 10.2527/jas1973.3751169x

[B37] Bazer FW Burghardt RC Johnson GA Spencer TE Wu G Interferons and progesterone for establishment and maintenance of pregnancy: interactions among novel cell signaling pathways Reprod Biol 2008 8 3 179 211 19092983 10.1016/s1642-431x(12)60012-6

[B38] Spencer TE Johnson GA Bazer FW Burghardt RC Fetal-maternal interactions during the establishment of pregnancy in ruminants Soc Reprod Fertil Suppl 2007 64 379 396 17491160 10.5661/rdr-vi-379

[B39] Spencer TE Johnson GA Bazer FW Burghardt RC Palmarini M Pregnancy recognition and conceptus implantation in domestic ruminants: roles of progesterone, interferons and endogenous retroviruses Reprod Fertil Dev 2007 19 1 65 78 10.1071/RD06102 17389136

[B40] Lenschow DJ Giannakopoulos NV Gunn LJ Johnston C O'Guin AK Schmidt RE Levine B Virgin HWt Identification of interferon-stimulated gene 15 as an antiviral molecule during Sindbis virus infection in vivo J Virol 2005 79 22 13974 13983 10.1128/JVI.79.22.13974-13983.2005 16254333 PMC1280211

[B41] Nakaya T Sato M Hata N Asagiri M Suemori H Noguchi S Tanaka N Taniguchi T Gene induction pathways mediated by distinct IRFs during viral infection Biochem Biophys Res Commun 2001 283 5 1150 1156 10.1006/bbrc.2001.4913 11355893

[B42] Herath S Lilly ST Fischer DP Williams EJ Dobson H Bryant CE Sheldon IM Bacterial lipopolysaccharide induces an endocrine switch from prostaglandin F_2α _to prostaglandin E_2 _in bovine endometrium Endocrinology 2009 150 4 1912 1920 10.1210/en.2008-1379 19056817 PMC2706387

[B43] Del Vecchio RP Matsas DJ Inzana TJ Sponenberg DP Lewis GS Effect of intrauterine bacterial infusions and subsequent endometritis on prostaglandin F_2α _metabolite concentrations in postpartum beef cows J Anim Sci 1992 70 10 3158 3162 1429292 10.2527/1992.70103158x

